# Evolutionary implications of the distribution and variation of the skeletal muscles of the anuran lymphatic system

**DOI:** 10.1007/s00435-013-0190-7

**Published:** 2013-04-03

**Authors:** Robert C. Drewes, Stanley S. Hillman, Michael S. Hedrick, Philip C. Withers

**Affiliations:** 1 Department of Herpetology, California Academy of Sciences, San Francisco, CA 94118 USA; 2 Department of Biology, Portland State University, Portland, OR 97207-0751 USA; 3 Department of Biological Sciences, University of North Texas, Denton, TX 76203 USA; 4 School of Animal Biology, University of Western Australia, Crawley, WA 6009 Australia

**Keywords:** Anuran lymphatic muscles, Phylogeny, Ancestral character states, Compliance model, Evolution

## Abstract

Lymphatic return to the circulation in anurans is dependent upon the interaction of a number of skeletal muscles and lung deflation. We define character states and describe variation of these putative lymphatic skeletal muscles: the M. cutaneus pectoris (CP), M. cutaneus dorsi (CD), M. piriformis (P), M. sphincter ani cloacalis (SAC), and the complex of the M. gracilis minor/M. abdominal crenator (GM/AC). We include examination of over 400 specimens of 377 species belonging to 40 of the 42 currently recognized anuran families. Some muscles show limited variation (P) or are clearly linked to phylogeny (CP; CD) and thus have limited value in the determination of form and function. However, the GM/AC and SAC show a high degree of structural variation that appears in taxa across the phylogenetic spectrum. This allows us to make phylogenetically independent determinations of form and function. We define an ancestral state of the GM and conclude that evolution of the GM/AC and SAC has progressed in two directions from this ancestral state: toward either elaboration or reduction. Where present, the character states of both of these muscle groups were observed in all species examined and the number of states correlated within each family as well. The degree of development of the GM/AC and SAC compliance pump system is strongly correlated with previously determined lymph flux rates in a three species test. Our data suggest there may be a relationship between greater elaboration of the GM/AC and SAC system and terrestriality among the Anura.

**Electronic supplementary material:**

The online version of this article (doi:10.1007/s00435-013-0190-7) contains supplementary material, which is available to authorized users.

## Introduction

The skeletal muscles putatively involved in lymph movement in anurans are the M. piriformis, the complex of M. gracilis minor and M. abdominal crenator, M. cutaneus dorsi, M. cutaneus pectoris, and M. sphincter ani cloacalis (Drewes et al. 2007). The basis for their involvement in the movement of lymph derives from (1) their insertion on or attachment to the skin dermis (except M. piriformis and M. sphincter ani cloacalis), (2) synchronized EMG activity between these muscles with both lung expiration and pressure differences recorded in subcutaneus lymph sacs sufficient to move lymph (Drewes et al. 2007; Hedrick et al. 2007), (3) compromised rates of lymphatic return to the circulation when the tendons of these muscles are cut (Hillman et al. 2010), and correlation of their presence and size with measured rates of lymph return to the circulation (Hillman et al. 2011). The notion that these skeletal muscles have a combined primary function in lymph movement is unique and not suggested in any previous description of their function. While this functional role may seem arcane to some investigators, lymphatic return is in fact physiologically vital for anurans.

**Fig. 1:**
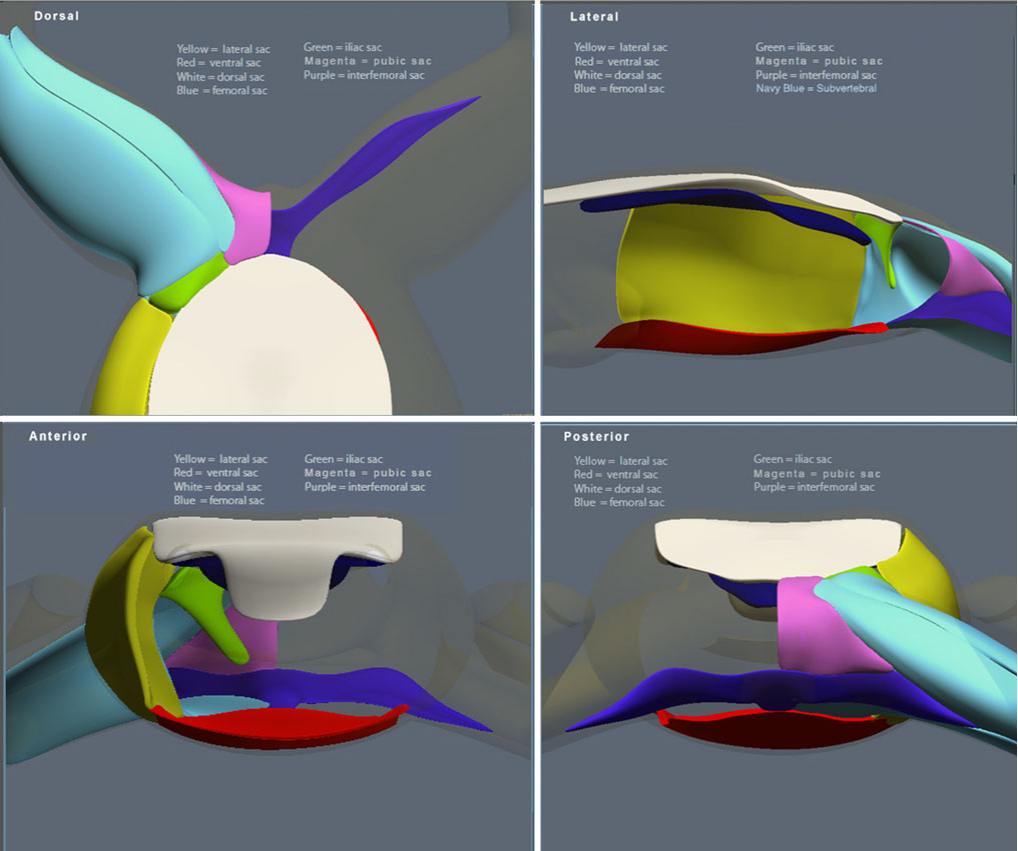
Stylized illustrations of location and orientation of typical anuran subcutaneus lymph sacs. Note that the subvertebral lymph sac is depicted, but it is not subcutaneus and thus not influenced by the lymphatic pump muscles discussed here

The role of the lymphatic system in all vertebrates is to return fluid lost from the capillaries to the circulatory system. Loss of blood volume (hypovolemia) leads to decline in cardiac output (hypovolemic shock) and death of the organism. The rate of capillary fluid loss (lymph formation) in anurans occurs at a rate up to ten times that of other vertebrates (Hillman et al. 2010). Anurans have extensive subcutaneus lymphatic sacs, and variation of these sacs has been described by Carter (1979). A schematic organization of the lymph sacs discussed in the manuscript is presented in Fig. 1. The morphological basis for the high rate of plasma turnover in anurans is the presence of these extensive subcutaneus lymph sacs, which create a large interstitial compliance (Hillman et al. 2010). The high interstitial compliance favors pooling of lymph in gravitationally dependent regions of the body (Hillman et al. 2004), the escape of plasma from the capillaries (Hancock et al. 2000; Hillman et al. 2010), and leads to high rates of plasma turnover—up to ten times that of mammals and birds per unit of tissue (Hillman et al. 2010). The pooling of lymph in dependent body regions is especially problematic for anurans. The lymphatic hearts which generate the pressure necessary to move the lymph back into the venous circulation are located on the dorsal surface of the animal (Kampmeier 1969). Hence, movement of the lymph dorsally from the ventral lymph sacs to the lymph hearts requires a physical driving force sufficient to overcome that of gravity. We have hypothesized that contraction of these skeletal muscles in conjunction with lung expiration generates the pressure difference required to move the lymph dorsally (Hillman et al. 2004). The goals of the current study were to evaluate the range of morphological variation present in each muscle and to preliminarily assess both the phylogenetic pattern and large-scale natural history association of this variation.

## Muscles involved in the compliance pump model

**Fig. 2:**
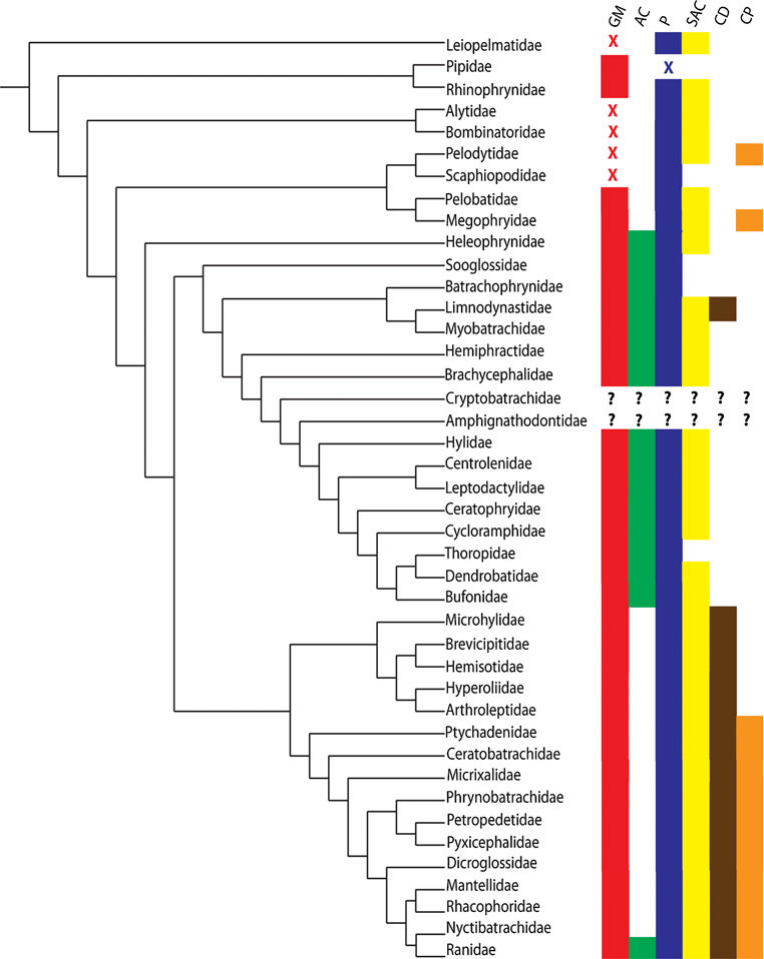
The presence of skeletal muscles involved in lymphatic movement among the families of the anurans: M. gracilis minor—*red*, X = State 4 (see text); M. abdominal crenator—*green*; M. piriformis—*blue*, X = absent or reduced); M. sphincter ani cloacalis—*yellow*; M. cutaneus dorsi—*brown*; M. cutaneus pectoris—*orange*. Cladogram adapted from Frost et al. (2006, Fig. 66)

In most anurans, there are six muscles that contract synchronously with lung expiration; in our proposed compliance pump model, these serve to generate the pressure necessary for lymphatic return to the circulatory system via the lymphatic hearts. The presence or absence of these muscles among the anuran families is depicted in Fig. 2. The muscle terminology is generally from Gaupp (1896), except for M. abdominal crenator (Winokur and Hillyard 1992).

### M. ischiocutaneus

Noble (1922) described this muscle and thought it represented a detached outer portion of the M. sphincter ani cloacalis. This muscle is present in only two genera of two families, the Rhinophrynidae and Scaphiopodidae. While no physiologic data exist to support a definite role in lymph movement, its origin (similar to the SAC) and cutaneus insertion (similar to M. cutaneus dorsi) suggest such a role. The M. ischiocutaneus is not included in this analysis.

### M. piriformis (Fig. 3a)

Our reinterpretation of the M. piriformis (Drewes et al. 2007) includes the assumption that its origin is on the femur and insertion is on the urostyle, so that when the M. piriformis contracts, the urostyle is depressed, directly changing the volume and pressure of the pubic lymph sac. This interpretation is supported by our finding that contraction of the M. piriformis muscles in *R. marina* and *L. catesbeianus* measured by electromyography (EMG) also causes movement of the urostyle when the animals are not moving (Drewes et al. 2007). This interpretation differs from every other description of the M. piriformis which assume its origin as the urostyle and insertion as the femur, inferring some function in leg elevation (Prikřyl et al. 2009) or by providing pelvic rigidity during locomotion (Emerson and De Jongh 1980; Reilly and Jorgensen 2010). In the majority of neobatrachian frog groups, the urostyle has a monocondylar or bicondylar joint with the sacrum (Trueb 1973), presumably allowing for ventral–dorsal movement. The union of the muscle with the urostyle is at the latter’s distal end, far removed from its articulation with the sacrum. Given the typical mono- or bicondylar joint, the long lever arm, and the mass differences between the hind limb and urostyle, it is difficult to support the hypothesis that the urostyle is the origin for the M. piriformis. We maintain that contraction of the M. piriformis depresses the urostyle (Drewes et al. 2007) and that the latter moves in a ventral–dorsal manner analogous to a pump handle. It is possible that the M. piriformis is also active during other functions such as locomotion, but a primary role as a locomotor muscle for elevating the femur is not supported in light of our EMG data (Drewes et al. 2007).

**Fig. 3:**
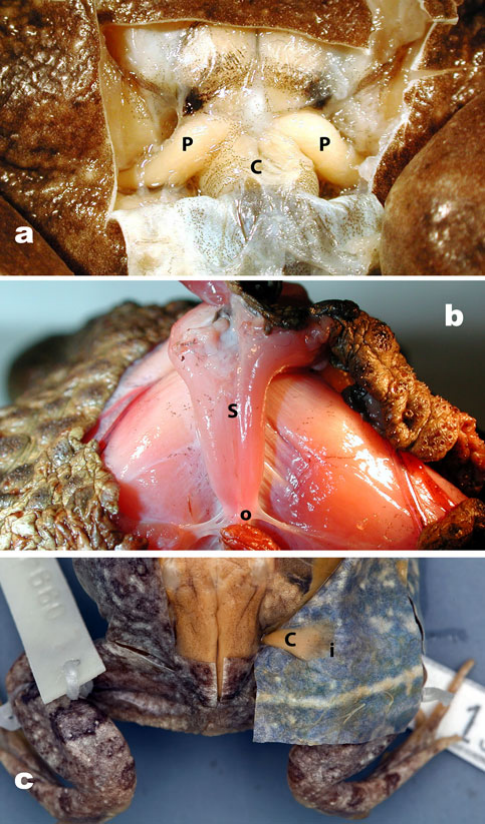
**a** Dorsal aspect of the insertions of the two symmetrical elements of the M. piriformis (*P*), dorsal view with dorsal skin reflected posteriorly to this insertion the M. sphincter ani cloacalis inserts on the cloacal tube (M. compressor cloacalis), (*C*) *Lithobates catesbeianus*—CAS 210381; **b** view of the posterior with M. sphincter ani cloacalis (*S*) in *Rhinella marina* (fresh, uncataloged). Origin (*o*) on fascia of the posterior rim of the pelvis *below*; this is also the origin of M. abdominal crenator (Fig 6b, c); *insertion* on dorsal surface of cloacal tube visible on **a** above; **c** dorsal view of insertion (*i*) of M. cutaneus dorsi (*C*) in *Tomopterna marmorata* (state 2—CAS 130883). Dorsal skin reflected posterolaterally to show insertion (*i*) along dorsal margin of the lateral lymph sac

### M. sphincter ani cloacalis (Fig. 3b)

This muscle was first described by Ecker (1864). The origin varies from the posterior-most apex of the pelvic rim to ventrally on the pubic symphysis (Drewes et al. 2007). The paired slips pass dorsally and tightly adhere to the dermis of the pubic sac; they meet and insert on the fibers of the M. compressor cloacalis on the dorsal rim of the cloaca. There is no formal description of function other than what can be inferred from its name, “a sphincter of the cloaca.” We maintain that contraction of this muscle in concert with the M. piriformis depresses the urostyle and at the same time influences the volume and pressure of the pubic lymphatic sac which is the principal ventral–dorsal pathway for lymph returning from the hind limbs.

### M. cutaneus dorsi (Fig. 3c)

This muscle originates on the connective tissue superficial to the ventral aspect of the pelvic disk and runs anteriorly and dorsally to an insertion on or along the dorsal margin of the lateral lymph sac close to the junction of dorsal, iliac, and femoral lymph sacs. It was described by Dugés (1834) as a tensor of the skin of the back. From a lymphatic perspective, contraction of this muscle will influence the volume and pressures of the lateral, iliac, and femoral lymph sacs. Its EMG activity is coincident with EMG activity of the M. piriformis and M. gracilis minor (Hedrick et al. 2007).

### M. cutaneus pectoris (Fig. 4a, b)

These paired strap-like muscles originate near the third inscription of the M. rectus abdominus, from the anterior edge of the most posterior extent of the metasternal portion of the pectoral girdle. The muscles pass anteriorly and are relatively uniform in width until their insertion on the posterior margin of the pectoral lymph sac. Gaupp (1896) was the first to suggest a possible role in lymph movement in abdominal and pectoral lymph sacs. Tyler (1971) suggested the presence of this muscle throughout the Ranidae (sensu lato), but Drewes (1984) demonstrated its absence in the Hyperoliidae and suggested this loss might allow for increased expansion of the vocal sac during advertisement calling in some groups. Drewes et al. (2007) stated that contraction of the muscle will pull the central portion of the pectoral lymph sac septum posteriorly, increasing pectoral pressure (decreasing volume) and subsequently decreasing abdominal lymph sac pressure (increasing volume).

**Fig. 4:**
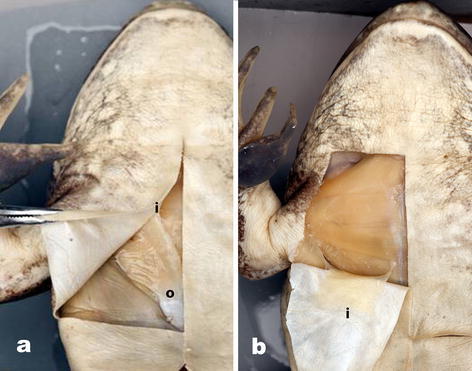
Ventral view of origin (*o*) and insertion (*i*) of M. cutaneus pectoris in *Lithobates catesbeianus* **a** CAS 210381, skin reflected obliquely to show origin (*o*) at the lateral margin of the metasternum, and **b** insertion (*i*) along the posterior margin of the pectoral lymph sac (state 2, specimen uncataloged)

### M. gracilis minor/M. abdominal crenator complex (Figs. 5a–c, 6a–c)

The M. gracilis minor is present in all anurans except the dendrobatid treefrogs of the genus *Phyllobates* (Dunlap 1960). Noble (1922) concludes “fossorial forms do tend to have greater expansion and attachment of the M. gracilis minor to the skin than do terrestrial or aquatic genera.” Dunlap (1960) examined 31 species of 15 anuran families (designations here are corrected to reflect those of Frost et al. (2006)) and tabulated three categories of the M. gracilis minor: (1) “Origin largely from pelvis” in one leiopelmatid (*Ascaphus*), two pipids, one scaphiopodid, a megophryid, a cycloramphid, a leptodactylid, a hylid, a centrolenelid, two ranids, and a microhylid; (2) “Origin largely from skin” in the other leiopelmatid genus, *Leiopelma,* a bombinatorid, and two alytids (but see GM/AC character state 4, Fig. 6a, below); and (3) “Origin by two heads” (“one from the ischiac region of the skin and the other from the skin”) in two bufonids, two limnodynastids, two leptodactylids, a cycloramphid, a phrynobatrachid, and two rhacophorids. This muscle has gone largely unexamined in detail since Dunlap (1960); it is, in fact, the most variable of all of our proposed compliance pump skeletal muscles. Our work suggests that Dunlap’s (1960) description of a two-headed origin may be the result of this variation and/or confusion with the M. abdominal crenator (Fig. 6b, c).

**Fig. 5:**
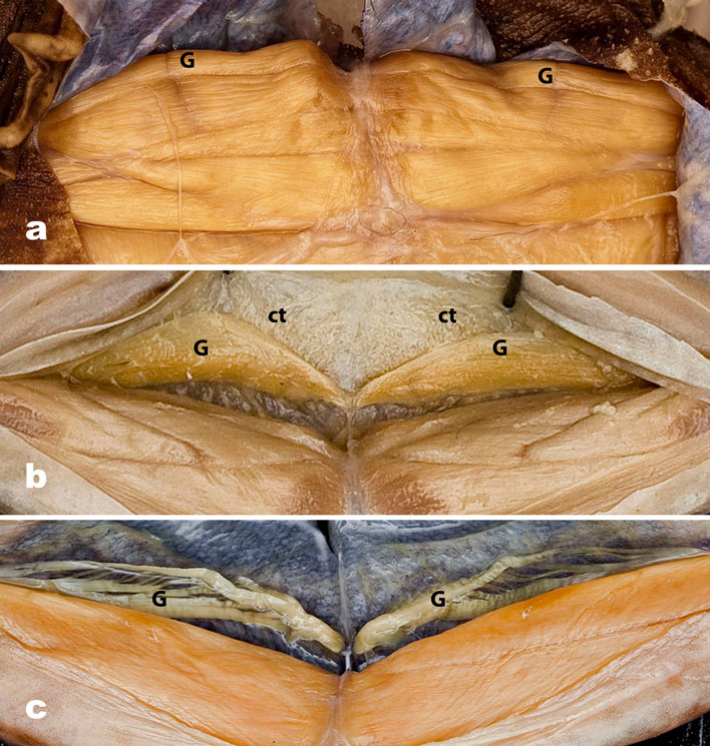
Variation in the M. gracilis minor (*G*)/M. abdominal crenator complex; all views are ventral with ventral skin reflected posteriorly; all specimens are oriented so that the top of each image is posterior. **a** State 1. *Pipa pipa* (CAS 93306), M. abdominal crenator is absent, M. gracilis minor (*G*) undifferentiated and unattached to skin of the thigh; **b** state 2. *Rana temporaria* (CAS 25826), M. abdominal crenator absent, and M. gracilis minor (*G*) undifferentiated but adherent by connective tissue fibers (*ct*) to skin of the thigh; **c** state 3. *Hylarana albolabris* (CAS 146100), M. abdominal crenator absent, and separate ventral muscles of the M. gracilis minor insert on the skin overlying the interfemoral lymph sac

**Fig. 6:**
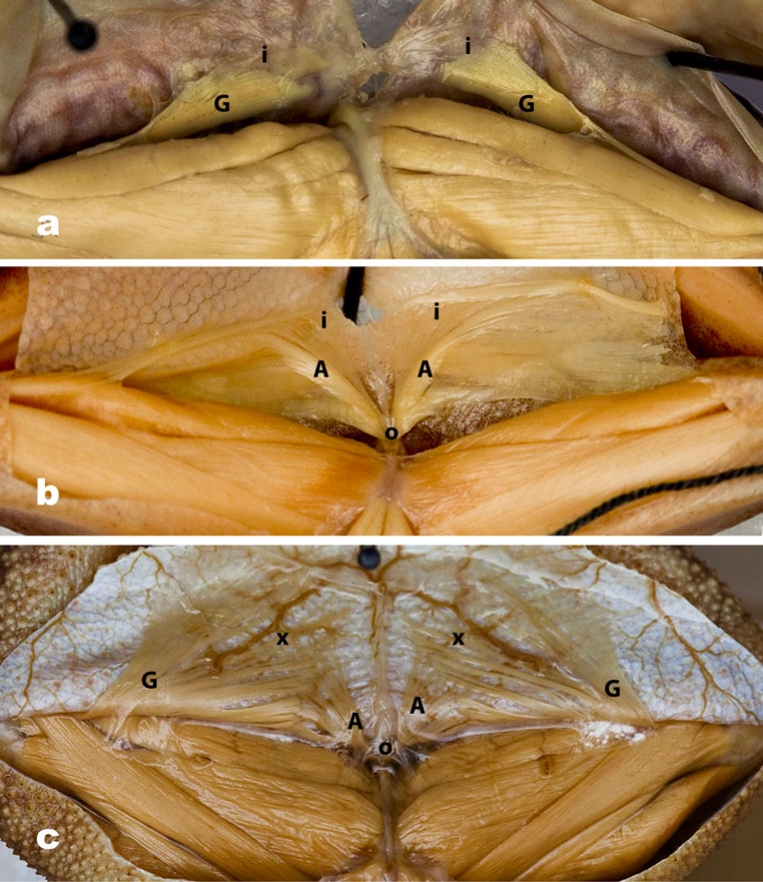
Variation in the M. gracilis minor (*G*)/M. abdominal crenator (*A*) complex; all views are ventral with ventral skin of the thigh reflected posteriorly; all specimens are oriented so that the top of each image is posterior. **a** State 4. *Scutiger sikimmensis* (CAS 90713), M. abdominal crenator absent, and all fibers of the M. gracilis minor insert (*i*) solely on the skin overlying the interfemoral lymph sac; the muscle does not reach the rim of the pelvis. **b** State 5. *Pseudacris regilla* (CAS–SU 13849), M. abdominal crenator present, originating (*o*) from pelvic rim, with minimal to moderate fanning and insertion (*i*) on the skin; M. gracilis minor has two components (see text). Origin of M. abdominal crenator and insertion of M. gracilis minor undifferentiated. **c** State 6. *Anaxyrus terrestris* (CAS–SU 8016) M. abdominal crenator (*A*) present, and its origin (*o*) well differentiated from insertion (*i*) of M. gracilis minor (see also Fig. 3b); fibers of both muscles broadly fanned and interdigitating at near right angles(x) (see text)

Previously, the M. abdominal crenator has been referred to as an “accessory head” of the M. gracilis minor (e.g., Cannatella 1985). It has since been more extensively examined in members of the family Bufonidae and described as a separate muscle by Winokur and Hillyard (1992). We suggest the functional role of this muscle is to vary the volume and pressure within the interfemoral lymph sac, which is the most gravitationally dependent lymph sac of the thigh and which communicates directly with the pubic lymph sac. The pubic sac is the dorsal–ventral corridor for lymph passage from the hind limb to the posterior lymph hearts. Contraction of the M. gracilis minor expands the volume and decreases the pressure of the interfemoral lymph sac drawing lymph from the femoral lymph sacs of the thighs. Contraction of the M. abdominal crenator compresses the interfemoral lymph sac, raising the pressure necessary to move lymph vertically through the pubic lymph sacs to the posterior lymph hearts (Drewes et al. 2007). Hence, elaboration of the insertion of heads of this complex should more completely expand and compress the interfemoral lymph sac, increasing the driving force and lymph flux from the ventral gravitationally dependent regions of the hind limbs to the posterior lymph hearts.

## Materials and methods

The morphologic variation in the muscles was determined by dissection of preserved specimens deposited in the collections of the Department of Herpetology, California Academy of Sciences as well as a number of other institutions. Over 400 individuals of 377 species were surveyed from 40 of the 42 families of anurans recognized in Frost et al. (2006) (material examined in Appendix 1). All museum acronyms in material examined follow Leviton et al. (1985). The phylogeny used to map this distribution is also that of Frost et al. (2006). We are aware that a number of well-supported lineages have since been recognized at the family level since Frost et al. (2006) but adhere to their 2006 phylogenetic construct (Fig. 2) to avoid errors in identification of material examined. Imagery was done by the BK 100 camera by Visionary Digital purchased under NSF IOS 0843082.

## Results

The presence of skeletal muscles involved in lymphatic movement among the families of the anurans presented below is illustrated in Fig. 2.

### Variation in M. piriformis

All extant anurans possess the M. piriformis with the exception of some members of the aquatic family Pipidae (Fig. 2). In those pipid frogs where it is present, it is much reduced in mass (Hillman et al. 2011). The insertions of the M. piriformis on the urostyle are illustrated in Fig. 3a.

### Variation in M. sphincter ani cloacalis

We define three character states of this muscle.

For all muscles, *State 0* Absent.

*State 1* Sphincter ani cloacalis originates on connective tissue tightly adhering the skin to the posterior-most margin of the pelvic rim (cartilago marginalis of Gaupp 1896) and inserts on the dorsal surface of the M. compressor cloacalis (Fig. 3a).

*State 2* Muscle origin is dorsal to state 1 more attached to the pelvic rim and the muscle is more massive than state 1 (Fig. 3b).

**Fig. 7:**
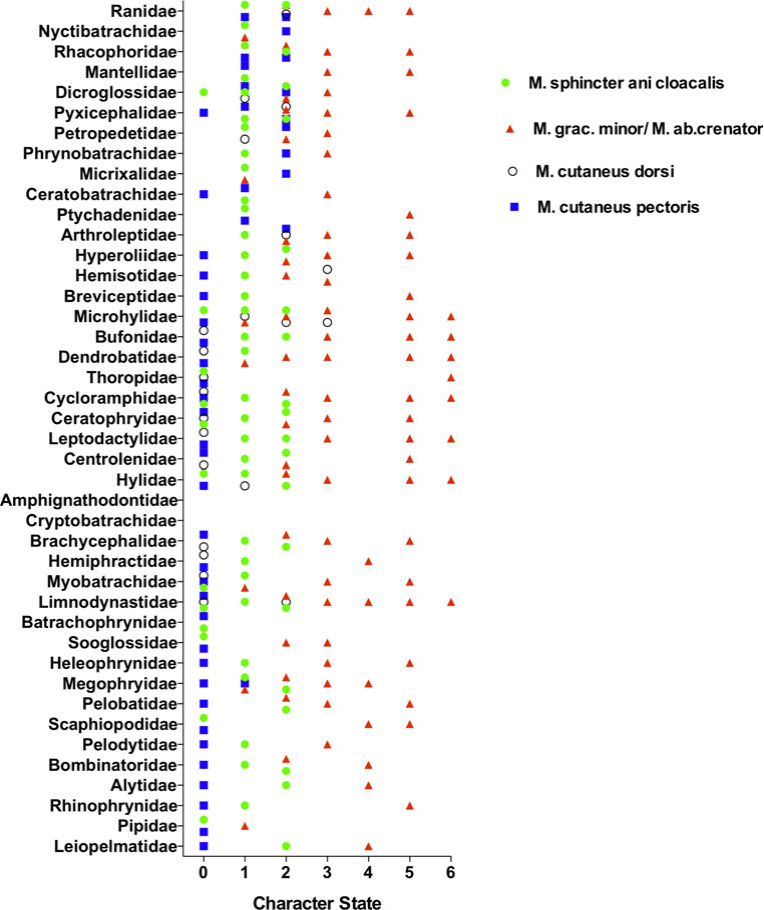
The phylogenetic characterization of various character states for the M. sphincter ani cloacalis, M. cutaneus dorsi, M. cutaneus pectoris, and M. gracilis minor/M. abdominal crenator

The distribution of character states of SAC is illustrated in Fig. 7. Fifteen of the 39 anuran families examined have multiple character states.

There is a significant correlation (*p* < 0.0001, Spearman *r* = 0.64) between number of character states contained within a family between the gracilis minor/abdominal crenator and sphincter ani cloacalis. At the species level, there is also a significant correlation between these two muscles (*p* < 0.0001, Spearman *r* = 0.31).

### Variation in the M. cutaneus dorsi

We define four character states of M. cutaneus dorsi:

- *State 0* The muscle is absent.
- *State 1* Width of insertion spanning less than 2 times its width at origin.
- *State 2* Width at insertion 2–3.5 times width at origin (Fig. 3c).
- *State 3* Width at insertion 4 times or greater than width at origin.

**Fig. 8:**
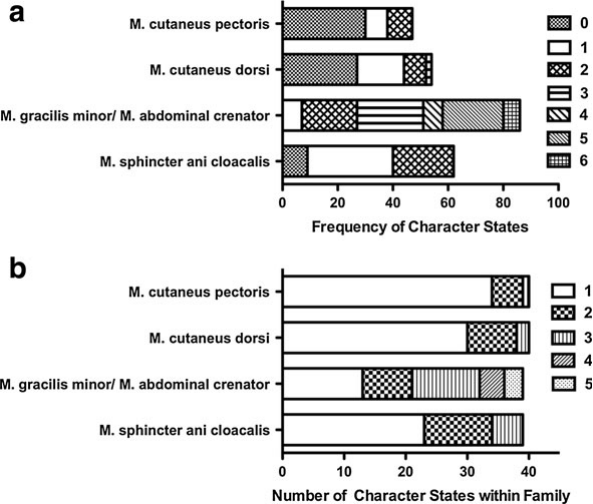
The frequency of various character states found in all the families (Fig. 7a) and the frequency of the number of character states contained in any family (Fig. 7b) for the M. sphincter ani cloacalis, M. cutaneus dorsi, M. cutaneus pectoris, and M. gracilis minor/M. abdominal crenator

The distribution of this skeletal muscle is limited to the Ranoidea (it is absent in the Hyloidea) with the exception of the six representatives of the Australian family Limnodynastidae (Fig. 2). There was no significant correlation at the species level between the M. cutaneus dorsi and either the gracilis minor/abdominal crenator complex (*p* = 0.65) or M. sphincter ani cloacalis (*p* = 0.44) (Fig. 8a–c).

### Variation in the M. cutaneus pectoris

The M. cutaneus pectoris can be divided into three character states.

- *State 0* The muscle is absent.
- *State 1* Muscle is present, width at insertion less than one-third the distance between the ventral midline and origin of the fore limb.
- *State 2* The width at insertion is twice or greater than the distance between the midline of the body and origin of the fore limb (Fig. 4a, b).

This muscle is only present in Ranoidea and the more basal Megophryidae and Pelodytidae (Fig. 2). At the species level, there were no significant correlations between the M. cutaneus pectoris and M. cutaneus dorsi (*p* = 0.34). M. gracilis minor/abdominal crenator (*p* = 0.11), and M. sphincter ani cloacalis (*p* = 0.13) (48 a, b, c).

### Variation in M. gracilis minor/M. abdominal crenator complex

Among derived families, this complex is comprised of two different skeletal muscles which, when both present, are coordinated in their activity as a single functional unit. This has physiological significance in large groups of anurans; thus, we assign character states irrespective of those cases in which one of these elements is always absent. We define six states of the complex:

- *State 1* The M. abdominal crenator is absent; the gracilis minor (G) is an unmodified thigh muscle running parallel to and overlying the gracilis major with no functional association with the skin, that is, inserting on the pelvic rim only (Fig. 5a).
- *State 2* The M. abdominal crenator is absent; the M. gracilis minor (G) is as in State 1 except that along its length there is connective tissue adherence to the skin overlying the margin of the interfemoral sac (Fig. 5b).
- *State 3* The M. abdominal crenator is absent; separate ventral muscle fibers of the M. gracilis minor insert on the skin of the interfemoral lymph sac. In this state, there are various degrees of elaboration of the M. gracilis minor (Fig. 5c).
- *State 4* The M. abdominal crenator is absent; in the M. gracilis minor, all fibers of the muscle insert solely on the skin overlying the interfemoral lymph sac. The M. gracilis minor does not reach the pelvic rim (Fig. 6a).
- *State 5* The M. abdominal crenator is present originating from the pelvic rim with minimal to moderate fanning on the skin overlying the interfemoral lymph sac. The M. gracilis minor has two components: Some fibers running continuously from the knee to the pelvic rim, but most fibers inserting on the skin perpendicular to the fibers of the abdominal crenator. The origin of the M. abdominal crenator and the insertion of the M. gracilis minor on the pubic rim are undifferentiated (Fig. 6b).
- *State 6* Separate origin of M abdominal crenator on the pelvic rim well differentiated from insertion of M. gracilis minor; the fibers of the abdominal crenator are greatly fanned and insert on the skin. The majority of the fibers of the gracilis minor insert on the skin and are likewise greatly fanned. They interdigitate at near right angles with the fibers of the abdominal crenator at their insertion overlying the interfemoral lymph sac. Some fibers of the M. gracilis minor remain continuous from the knee to insertion on pelvic rim (Fig. 6c).

There is a broad range of variation of the M. gracilis minor/M. abdominal crenator complex character states among the anuran families examined (Fig. 8a). The frequency of the character states within each family is illustrated in Fig. 8b. We suggest that the plesiomorphic state of this complex is state 4, which has a low frequency of occurrence but is widely present in the most basal lineages and rare among derived groups (Fig. 2). Seventeen of the 39 anuran families examined have three or more character states within them (Fig. 8b), and 26 of the 39 have multiple character states within them.

## Discussion

In an attempt to achieve as broad a phylogenetic perspective as possible, our study includes 377 species of 40 of the 42 families recognized by Frost et al. (2006); it represents the largest if not the only analysis of these muscles. However, we include only about 8 % of the currently described extant species and thus recognize that there may well be far more variation than we have described.

Many of the skeletal muscles examined that participate in the movement of lymph show a wide variation in size, mass, and insertional patterns. For a number of these muscles, no function has been previously proposed.

The amount of variation in size and configuration of a number of the muscles studied was unexpected. However, much of our knowledge of anuran myology is derived from the multiple and combined works of Alexander Ecker, Robert Wiedersheim, and Ernst Gaupp (1864–1904) which were wholly based on two species of the genus *Pelophylax* (*Rana*) both of which exhibit state 2 of the M. gracilis minor. This muscle has generally been overlooked in terms of alternative function beyond locomotory (except Winokur and Hillyard 1992). In fact, the family Ranidae actually includes four of the six different and distinct states of this muscle, some quite striking and clearly not related to locomotion.

Some of the muscles described above in our compliance pump model (see also Hillman et al. 2004) show either limited variation (M. piriformis) or are clearly distributed with phylogenetic bias (M. cutaneus pectoris, M. cutaneus dorsi) and thus have limited value in the determination of form and function. However, there are muscles that show a high degree of structural variation that appear across the anuran phylogenetic spectrum. These allow us to make phylogenetically independent determinations of form and function.

Based on our data, the most robust model with which to test form and function is the highly correlated variations in the M. gracilis minor/M. abdominal crenator complex and the M. sphincter ani cloacalis, since the relationship occurs across multiple lineages. Our synthesis of this variation suggests that the ancestral condition of the complex includes state 4 of the M. gracilis minor (Fig. 6a), since this state occurs mainly among the most basal extant lineages. In addition, the correlation reported above suggests that the ancestral state of the M. sphincter ani cloacalis is state 1. This would indicate that evolution of this system has been bidirectional and has included both the loss and elaboration of the muscles involved.

Functionally, we interpret increased insertion width and numbers of heads as enabling a more dispersed transmission of force to the skin and more effective compression of the attendant lymph sac. This would increase the pressure in the lymphatic sac increasing the rate of lymph movement from that sac. Data exist for the rate of lymph mobilization from the femoral lymphatic sac for three species: *Rhinella marina, Lithobates catesbeianus,* and *Xenopus laevis* (Hillman et al. 2011). Lymph moves from the femoral lymph sac to the interfemoral to the pubic lymph sac to ultimately reach the posterior lymph hearts (Hillman et al. 2004). The M. gracilis minor/M. abdominal crenator complex (GM/AC) and the M. sphincter ani cloacalis (SAC) play a major role in generating the force necessary to move the lymph from the femoral sac dorsally to the posterior lymph hearts. The rate of lymph flux in the three species is *R. marina* > *L. catesbeianus* > *X. laevis.* These flux rates are correlated with the degree of development of the muscles in the GM/AC and SAC: *R. marina* (GM/AC 6, SAC 3) > *L. catesbeianus* (2,1) > *X. laevis* (1,0) (Hillman et al. 2011). These data indicate that elaboration of these muscles from the presumptive ancestral state results in increased lymphatic flux, while loss or reduction of the muscles results in a decreased capacity to move lymph. These three species span the environmental gradient from terrestrial to aquatic.

Another way to test this statement is to analyze variation within anuran families exhibiting multiple states of these characters in order to determine whether there is an environmental correlation. Four anuran families show wide variation for both the GM/AC and SAC: Megophryidae, Limnodynastidae, Hylidae, and Microhylidae. Among the Megophryidae, the lowest character state scores for the GM/AC and SAC are found in the genus *Oreolalax* (1,1); the highest are found in *Scutiger sikimmensis* (4,2). Both appear to inhabit similar riparian, forested habitats. In the Limnodynastidae, the lowest scores occur in *Limnodynastes tasmaniensis* (2,1) and the highest in *Platyplectrum ornatum* and *Notaden melanoscaphus* (5,3; 6,1). *L. tasmaniensis* is an inhabitant of riparian habitats; *P. ornatum* and *N. melanoscaphus* are both burrowing forms. The hylid species with lowest scores is *Litoria moorei* (3,1) and the highest is *Corythomantis greening* (6,3). *L. moorei* is mainly aquatic while *C. greeningi* is scansorial in dry savannah conditions. Among the microhylid frogs examined, the lowest scores are found in *Chaperina fusca* (1,1) and the highest in *Kalophrynus pleurostigma* (6,2). *C. fusca* inhabits rainforest habitats, while *K. pleurostigma* is leaf-litter terrestrial form in drier habitats. These results are suggestive but not conclusive with respect to any correlation of the development of these muscles and habitat.

Several of the muscles included in this study have distributions that indicate a phylogenetic bias. These include the M. cutaneus pectoris and the M. cutaneus dorsi. Presence of character state 3 of the M. cutaneus dorsi only occurs in the families Hemisotidae and Microhylidae. The variation found in the M. cutaneus pectoris does not appear to be related to the ecology of the species possessing it.

This study represents the first attempt to extensively characterize the range of variation present in these lymphatic pump skeletal muscles. We suggest that the amount of variation present in these lymphatic skeletal muscles should provide a fruitful model to delineate the environmental selective pressures responsible for creating functional differences in mobilizing lymph from the posterior regions of anurans. Such an analysis requires the ability to objectively characterize the ecology and correct for phylogenetic constraints for multiple species. Characterizing the range of variation present in each of these muscles represents the first step in building such a dataset.

## Electronic supplementary material

Below is the link to the electronic supplementary material.

Supplementary material 1 (DOC 158 kb)
