# Supplementary material for: Evolutionary implications of the distribution and variation of the skeletal muscles of the anuran lymphatic system
Source: Zoomorphology. 2013 Apr 3;132(3):339–49. doi: 10.1007/s00435-013-0190-7 (PMC3742416; doi:10.1007/s00435-013-0190-7)
Supplement: Supplementary file 1 — Supplementary material 1 (DOC 158 kb) [file 435_2013_190_MOESM1_ESM.doc]

**Appendix I.**

Evolutionary implications of the distribution and variation of the skeletal muscles of the anuran lymphatic system.

Zoomorphology. Robert C. Drewes, California Academy of Sciences [rdrewes@calacademy.org](mailto:rdrewes@calacademy.org), Stanley S. Hillman, Michael S.Hedrick, Philip C. Withers

**Material Examined.**

Family names and species content follow Frost (2006); some generic names may have since been changed; these reflect the names under which they were housed at CAS or borrowed from other institutions**.**

**Alytidae:** *Alytes obstetricians* CAS-SU 21691, Discoglossus pictus CAS 138451, *Discoglossus sardus* CAS-SU 19649;

**Arthroleptidae:** *Arthroleptis variabilis* CAS 199159, *Astylosternus montanus* CAS 125586, *Cardioglossa leucomystax* CAS 196116, *Leptopelis bocagei* CAS 141448*, Leptopelis flavomaculatus* CAS 153639, *Leptopelis vermiculatus* CAS 162508, *Leptopelis viridis* CAS 97543, *Nyctibates corrugatus* CAS 153797, *Schoutedenella schubotzi* CAS 201755, *Scotobleps gabonicus* CAS 153579, *Trichobatrachus robustus* CAS 38844;

**Batrachophrynidae:** *Calyptocephalella gayi* (*C. caudiverbera*) USNM 139788;

**Brachycephalidae:** *Barycholos pulcher* CAS-SU 10498, *Brachycephalus ephippium* CAS 94872, *Bryophryne cophites* KU 173301, *Craugastor augusti* CAS 65891, *Craugastor rugulosus* CAS 143084, CAS 101377, *Ischnocnema guentheri* CAS 11894; *Lynchius parkeri* KU 181321, *Oreobates quixensis* CAS-SU 11454, *Pleurodema thaul* CAS 86675, *Pristimantis achatinus* CAS-SU 10608, *Pristimantis chloronotus* CAS-SU 17412, *Silverstoneia nubicola flotator* CAS 98332, *Strabomantis anomalus* CAS 119755;

**Bombinatoridae:** *Barbourula busuangensis* CAS-SU 21240, *Bombina bombina* CAS 144396, *Bombina maxima* CAS 194886, *Bombina orientalis* CAS 32511, *Bombina variegata* CAS 164372;

**Brevicipitidae:** *Breviceps adspersus* CAS 200131, *Breviceps gibbosus* CAS 170327, *Breviceps macrops* CAS 165324, *Callulina kreffti* CAS 168717;

**Bufonidae:** *Amietophrynus camerunensis* CAS 207290, *Amietophrynus kisoloensis* CAS 202216, *Amietophrynus regularis* CAS 153188, *Anaxyrus americanus* CAS 207257, *Anaxyrus boreas* CAS 209391, *Anaxyrus canorus* CAS 97683, *Anaxyrus cognatus* CAS 188305, *Anaxyrus debilis* CAS 171994, *Anaxyrus hemiophrys* CAS 54750, *Anaxyrus punctatus* CAS 31359, *Anaxyrus terrestris* CAS-SU 8022 , *Anaxyrus woodhousii* CAS-SU 7627, *Ansonia muelleri* CAS -SU 19258, *Atelopus ignescens* CAS 152058 *Bufo bufo* CAS 21702 *Bufo macrotis* CAS 230357 *Bufo pentoni* CAS 136200 *Dendrophryniscus brevipollicatus* CAS 93819, *Didynamipus sjostedti* CAS 162676, *Duttaphrynus melanostictus* CAS 221799, *Incilius alvarius* CAS 92474, *Incilius bocourti* CAS 70702, *Incilius coniferus* CAS 119450, *Incilius fastidiosus* CAS 178160, *Incilius macrocristatus* CAS 163782, *Incilius mazatlanensis* CAS 99639, *Incilius tacanensis* CAS 139890, *Incilius valliceps* CAS 73580, *Leptophryne borbonica* FMNH 185794, *Mertensophryne micranotis* CAS 160960, *Nannophryne cophotis*  KU 211693, *Nectophryne afra* CAS 103329, *Nectophrynoides tornieri* CAS 161962, *Nectophrynoides viviparous* CAS 202677, *Pedostibes hosei* CAS 105986, *Phrynoides aspera* CAS-SU 6722, *Poyntonophrynus lughensis* CAS 130549, *Pseudepidalea viridis* CAS 139948, *Rhaebo glaberrimus* CAS-SU 10335, *Rhaebo haematicus* CAS 94752, *Rhinella arenarum* CAS-SU 11990, *Rhinella festae* CAS-SU 11417, *Rhinella marina* CAS-SU 16992, *Rhinella ocellatus* CAS-SU 8402, *Schismaderma carens* CAS 151193, *Ingerophrynus biporcatus philippinicus* CAS 157224, *Ingerophrynus macrotis* CAS 174110, *Pelophryne lighti* CAS-SU 19712, *Peltophryne peltocephalus* CAS-SU 7769, *Vandijkophrynus gariepensis* CAS 200136;

**Centrolenidae:** *Allophryne ruthveni* KU 166719, *Centrolene prosoblepon* CAS-SU 10564; H*yalinobatrachium fleischmanni* CAS 69740;

**Ceratobatrachidae:** *Batrachylodes elegans* CAS 191186, *Batrachylodes vertebralis* CAS-SU 21808, *Batrachylodes wolfi* CAS-SU 23982, *Ceratobatrachus guentheri* CAS 135222, *Discodeles bufoniformis* CAS 109873, *Discodeles guppyi* CAS-SU 9344, *Palmatorappia solomonis* CAS 107478, *Platymantis dorsalis* CAS 137651, *Platymantis vitianus* CAS 172532, CAS 172453, *Telmatobius culeus* FMNH 203676;

**Ceratophryidae:** *Atelognathus patagonicus* CAS 100501, *Batrachophrynus macrostomus* CAS 74601, *Batrachyla taeniata* CAS 85254, *Ceratophrys aurita,* CAS-SU 11224, *Ceratophrys cranwelli* CAS-SU 11257, *Lepidobatrachus laevis* CAS uncat., *Telmatobius hauthali* CAS-SU 11266;

**Cycloramphidae:** *Alsodes vanzolinii* KU 162222, *Crossodactylus gaudichaudii* CAS-SU 11777, *Cycloramphus fuliginosus* CAS-SU 11701, *Eupsophus roseus* CAS 84736, *Odontophrynus americanus* CAS 100485, *Proceratophrys cristiceps,* CAS-SU 11931, *Rhinoderma darwinii* CAS 84738;

**Dendrobatidae:** *Allobates kingsburyi* CAS-SU 10346, *Ameerega picta* CAS-SU 11865, *Ameerega trivittatus* CAS-SU 11871, *Colostethus inguinalis* CAS 98305, *Dendrobates auratus* CAS 98412, *Dendrobates histrionicus* CAS 119265, *Hyloxalus elachyhistus* CAS 93895;

**Dicroglossidae:** *Altirana parkeri* CAS 177775, *Euphlyctis cyanophlyctis* CAS 243593, *Fejervarya cancrivora* CAS 124271, CAS 124268, *Hoplobatrachus occipitalis* , CAS 97519, *Hoplobatrachus tigerinus* CAS-SU 9095, *Limnonectes acanthi* CAS-SU 5988, *Limnonectes kuhlii* CAS 172703, *Occidozyga laevis* CAS 137309, *Quasipaa boulengeri* FMNH 19031;

**Heleophrynidae:** *Hadromophryne natalensis* CAS 156302, *Heleophryne purcelli* CAS 157090;

**Hemiphractidae:** *Gastrotheca riobambae* CAS 152041, *Hemiphractus proboscidea* CAS 122209;

**Hemisotidae:** *Hemisus guineensis* CAS-SU 13014, *Hemisus marmoratum* CAS 103694;

**Hylidae:** *Acris crepitans* CAS-SU 7895, *Agalychnis callidryas* CAS 142774, *Anotheca spinosa* CAS 85680, *Aparasphenodon brunoi* CAS-SU 12646, *Corythomantis greening* KU 125380, *Cyclorana novaehollandiae* CAS 77828, *Dendropsophus microcephala* CAS 143887, *Duellmanohyla rufioculis* CAS 122678, *Exerodonta chimalapa* CAS 163309, *Exerodonta smaragdina* CAS 92007, *Exerodonta surmichrasti* CAS 143140, *Hyla arborea* CAS 164396, *Hyla arenicolor*  CAS 95874, *Hyla cinerea* CAS 90052, *Hyla gratiosa* CAS 169438, *Hyla japonica* CAS 35847, *Hyla squirella* CAS 64950, *Hyla versicolor* CAS-SU 10884, *Hypsiboas albomarginatus* CAS-SU 12600, *Isthmohyla pictipes* CAS 122656, *Litoria alboguttata* CAS 77977, *Litoria aurea*  CAS 162170, *Litoria bicolor* CAS 49195, *Litoria caerulea* CAS 121506, *Litoria gracilenta* CAS 116823, *Litoria meiriana* AMNH 126159, *Litoria moorei* CAS 78638, *Litoria nasuta* CAS 121278, *Litoria platycephalus* AMNH 152880, *Litoria rubella* CAS 82833, *Osteocephalus taurinus* CAS 49773, *Osteopilus septentrionalis* CAS 111040, *Pachymedusa dacnicolor* CAS 93856, *Phrynohyas venulosa*  CAS-SU 12307, *Phyllomedusa hypochondrialis* CAS 94839, *Phyllomedusa sauvagii* CAS-SU 11467, *Plectrohyla chryses* CAS 142943, *Pseudacris crucifer* CAS 71846, *Pseudacris regilla* CAS-SU 13849, *Pseudacris triseriata*  CAS 54744, *Pseudis limellus* CAS-SU 11632, *Pternohyla fodiens* CAS 94262, 99458, *Ptychohyla bistincta* CAS 199833, *Scinax ruber* CAS 152205, *Scinax x-signatus* CAS-SU 12432, *Smilisca baudinii* CAS 95669, *Smilisca dentata* CAS 135057, *Sphaenorhynchus lacteus* CAS-SU 12641, *Triprion spatulatus* CAS 142483;

**Hyperoliidae:** *Acanthixalus spinosus* CAS 153800, *Afrixalus dorsalis* CAS 207523, *Afrixalus fornasinii* CAS 184192, *Callixalus pictus* CAS 145261, *Chryobatrachus cupreonitens* CAS 145263*, Cryptothylax greshoffi* CAS 153613, 153614, *Heterixalus boettgeri* CAS 186109, *Hyperolius argus* CAS 164640, CAS 164654, *Hyperolius horstocki* CAS 157097, *Hyperolius kivuensis* CAS 180089, *Hyperolius marmoratus* CAS 156286, *Hyperolius molleri* CAS 218876, *Hyperolius nasutus* CAS 197376, *Hyperolius parkeri* CAS 164948, *Hyperolius thomensis* CAS 218925, *Hyperolius viridiflavus* CAS 164553, *Kassina cochranae* CAS 136257, *Kassina fusca* CAS 136217, *Kassina kuvangensis* CAS 196722, *Kassina parkeri* CAS 140361*, Kassina senegalensis* CAS 141662, *Kassinula wittei* CAS 196674, *Paracassina kouhniensis* CAS 145356, *Phlyctimantis verrucosus* CAS 176951, *Semnodactylus wealei* CAS 170383, *Tachynemis seychellensis* CAS 157507;

**Leiopelmatidae:** *Ascaphus truei* CAS 64789, *Leiopelma hochstetteri* CAS-SU 6709;

**Leptodactylidae:** *Edalorhina perezi* CAS 162132, *Eleutherodactylus coqui* CAS 141952*, Eleutherodactylus marnockii* CAS-SU 11202, *Engystomops pustulatus* CAS-SU 9499, *Leptodactylus bufonius* CAS-SU 11510, *Leptodactylus chaquensis* CAS-SU 11502, *Leptodactylus ocellatus* CAS-SU 11851, *Leptodactylus pentadactylus* CAS-SU 10454, *Physalaemus centralis* CAS-SU 12858, *Pleurodema fuscomaculatum* CAS-SU 12840;

**Limnodynastidae:** *Adelotus brevis* CAS 82351, *Heleioporus albopunctatus* CAS 77980, *Lechriodus fletcheri* CAS 82231, CAS 82371, *Limnodynastes convexiusculus* CAS 121263, *Limnodynastes dumerilii* CAS 82197, *Limnodynastes lignarius* AMNH 97339, *Limnodynastes peronii* CAS 82059, *Limnodynastes salmini* CAS 82125, *Limnodynastes tasmaniensis* CAS 82132, *Neobatrachus pictus* CAS-SU 10013*, Notaden bennettii* CAS 82688, *Notaden melanoscaphus* AMNH 38868, *Notaden nichollsi* AMNH 51654, *Philoria sphagnicolus* AMNH 139084, *Platyplectrum ornatum* CAS 78043, *Platyplectrum spenceri* AMNH 120241;

**Mantellidae:** *Boophis tephraeomystax* CAS 156883, *Laliostoma labrosus* CAS 86732, *Mantella betsileo* CAS 156767, *Mantidactylus* *ulcerosus* CAS 63949;

**Megophryidae** *Leptobrachium hasselti* CAS-SU 22296, *Megophrys montana stejnegeri* CAS-SU 21935, *Oreolalax pingii* CAS 194560, *Oreolalax rugosus* CAS 194842, *Scutiger boulengeri* CAS 177840*, Scutiger sikimmensis* CAS 90713;

**Micrixalidae:** *Micrixalus borealis* CAS 223299;

**Microhylidae:** *Albericus darlingtoni* CAS 108711, *Austrochaperina adelphe* AMNH 97898, *Austrochaperina almipes* CAS 154155, *Calluella guttulata* CAS 73733, *Chaperina fusca* CAS-SU 21546, *Cophixalus riparius* CAS 108277, *Dermatonotus muelleri* CAS-SU 11560, *Elachiostocleis ovalis* CAS-SU 11564, *Gastrophryne olivacea* CAS 174097,

*Glyphoglossus molossus* CAS 174132, *Hamptophryne boliviana* KU 150635*, Hylophorbus rufescens rufescens* CAS 154326, *Hypopachus barberi* CAS 139881, *Hypopachus variolosus* CAS 140982, *Kalophrynus pleurostigma* CAS-SU 17151, *Kaloula picta* CAS-SU 17530, *Microhyla berdmorei* CAS 216537, *Micryletta inornata* CAS 123847; *Nelsonophryne aequatorialis* KU 178312, *Oreophryne anulata* CAS 64087, *Phrynomantis wilhelmana* CAS 127395, *Phrynomantis bifasciatus* CAS 164886, *Probreviceps macrodactylus* CAS 168666, *Sphenophryne brevicrus* CAS 117193.

**Myobatrachidae:** *Assa darlingtoni* AMNH 80524, *Crinia bilingua* AMNH 140201, *Crinia insignifera* CAS 78090, *Crinia signifera* CAS 78655, *Mixophyes fasciolatus* CAS 82049, *Myobatrachus sp.*AMNH 39171, *Paracrinia haswelli* AMNH 171040 , *Pseudophryne bibronii* CAS 78105, *Pseudophryne guntheri* CAS 95246, *Pseudophryne occidentalis* CAS 100954, *Rheobatrachus silus* CAS 153753, *Taudactylus acutirostris* FMNH 225874, *Taudactylus diurnus* FMNH 226077, *Uperoleia laevigata* CAS 82668, *Uperoleia rugosa* CAS 82587, 82664;

**Nyctibatrachidae:** *Nyctibatrachus major* CAS 125376;

**Pelobatidae:** *Pelobates cultripes* CAS 156259, *Pelobates fuscus* CAS 173214, *Pelobates syriacus* CAS 223779, *Pelobates varaldi* CAS-SU 20378;

**Pelodytidae:** *Pelodytes caucasicus* CAS 94060;

**Petropedetidae:** *Arthroleptides martiensseni* CAS 168625, *Conraua crassipes* CAS 103909, *Conraua goliath* CAS uncat, *Indirana beddommii*  CAS 94971, *Indirana leithii* CAS 104288, *Petropedetes newtoni* CAS 125585;

**Phrynobatrachidae:** *Phrynobatrachus natalensis* CAS 154428, *Phrynobatrachus plicatus* CAS 136305;

**Pipidae:** *Hymenochirus boettgeri* CAS 98190, *Hymenochirus curtipes* CAS-SU 12958, *Pipa* carvalhoi CAS-SU 11595, *Pipa pipa* CAS 93306, *Pseudhymenochirus merlini* CAS 230132, *Xenopus fraseri* CAS 153596, *Xenopus laevis* CAS 220110, *Xenopus tropicalis* CAS 123522;

**Ptychadenidae:** *Hildebrandtia ornate,* CAS 154658, *Ptychadena anchietae*, CAS 154387;

**Pyxicephalidae:** *Amietia wittei* CAS 155075, *Arthroleptella hewitti* CAS 157245, *Aubria subsigillata* CAS 144215, *Cacosternum boettgeri* CAS 125890, *Cacosternum namaquense* CAS 193528, *Microbatrachella capensis* CAS 157008, *Natalobatrachus bonebergi* CAS 15724, *Nothophryne broadleyi* CAS 156127, *Pyxicephalus adspersus* CAS 183218, CAS 164710, *Pyxicephalus edulis*, CAS 164710, *Strongylopus grayii* CAS 211668, *Tomopterna breviceps* CAS 231407, *Tomopterna marmorata* CAS 130583;

**Ranidae:** *Amolops viridimaculatus* CAS 234063, *Babina okinavani* CAS 22833, *Babina pleuraden* CAS 242526, *Clinotarsus alticola* CAS 225162, *Glandirana rugosa* CAS 32653, *Huia cavitympanum* FMNH 146192, *Hylarana albolabris* CAS 146101, CAS 207653, *Hylarana chalconota*  CAS 229611, *Lithobates capito* USNM 129951, *Lithobates clamitans* CAS-SU 5520, *Lithobates grylio* CAS-SU 6746, *Lithobates palmipes* CAS 93351, *Lithobates septentionalis* CAS-SU 7216, *Lithobates esculenta* CAS 223790, *Lithobates sphenocephala* CAS-SU 8908, *Lithobates sylvatica* CAS 39703, *Lithobates pipiens* CAS 159155, *Meristogenys jerboa* CAS-SU 7288, *Odorrana andersonii* CAS 234026, *Odorrana schmackeri* CAS 194357, *Pelophylax lessonae* USNM 576009, 576010, *Pelophylax porosus* CAS 135850, *Pelophylax ridibunda* CAS 217852, *Pterorana khare* CAS 235190, *Rana cascadae* CAS 13304, *Lithobates catesbeiana* CAS 210381, *Rana muscosa* CAS 27408, *Rana temporaria* CAS 25825, *Sanguirana sanguinea* CAS-SU 21282, *Staurois natator* CAS 172882;

**Rhacophoridae:** *Buergeria japonica* CAS 211463, *Chirixalus effingeri* CAS 211458, *Chirixalus idiootocus* CAS 211525, *Chiromantis petersi* CAS 130640; 152834, *Chiromantis rufescens* CAS 207604, *Chiromantis xerampelina* CAS 164882, *Nyctixalus spinosus* CAS 139319, *Philautus emembranatus* CAS 183415, *Philautus poecilus* CAS 210862, *Philautus shmackeri* CAS-SU 22690, *Polypedetes leucomystax* CAS-SU 16226, CAS-SU 11113, *Rhacophorus arboreus* CAS 211265;

**Rhinophrynidae:** *Rhinophrynus dorsalis* CAS 138045;

**Scaphiopodidae:** *Scaphiopus couchii* CAS-SU 15663, CAS 188257, *Scaphiopus holbrooki* CAS162802, *Spea bombifrons* CAS-SU 7431, CAS-SU 17291, *Spea hammondii* CAS 233765, *Spea intermontana* CAS-SU 1885, *Spea multiplicata* CAS 188123;

**Sooglossidae:** *Nesomantis thomasetti* CAS 160083, *Sooglossus gardineri* CAS 156988;

**Thoropidae**: *Thoropa miliaris* CAS-SU 11736;
